# Comparison of the Physicochemical, Rheological and Functional Properties of Chicken Feet Gelatin Extracted by Acidic and Microwave Methods and Commercial Bovine Gelatin

**DOI:** 10.1002/fsn3.70651

**Published:** 2025-07-18

**Authors:** Hanieh Esmaeili‐Kaliji, Reza Farahmandfar, Ali Motamedzadegan, Maryam Asnaashari

**Affiliations:** ^1^ Department of Food Science and Technology Sari Agricultural Sciences and Natural Resources University (SANRU) Sari Iran; ^2^ Department of Animal Processing Animal Science Research Institute of Iran (ASRI), Agricultural Research, Education and Extension Organization (AREEO) Karaj Iran

**Keywords:** chicken feet gelatin, FTIR, microwave, quality properties

## Abstract

The poultry industry produces many by‐products such as chicken feet. These by‐products contain a relatively high amount of protein, especially collagen, which is effective for producing gelatin. Gelatin is a biopolymer with unique properties widely used in food and pharmaceutical industries. This study aimed to determine the physicochemical properties of chicken feet gelatin extracted using acidic and microwave treatment, and compared them with a commercial bovine gelatin. The results showed color (L*, a*, b*), gel strength, viscosity, turbidity, gelling and melting temperature, and amino acid composition of chicken feet gelatin were significantly higher quality than those of the commercial one. FTIR analysis showed all the amide bands in acidic gelatin and microwave‐extracted gelatin, but not in commercial bovine gelatin (band B). In temperature scanning and gel formation analysis, the highest melting temperature and gel formation were related to gelatin extracted by acidic treatment. Moreover, the gelatin obtained through microwave extraction exhibited improved quality compared to acidic extraction, showing higher viscosity and gel strength. The microscopic structure of microwave‐extracted gelatin compared to acidic and bovine gelatin had numerous holes and showed the highest turbidity. The structural stability of chicken feet gelatin is more potent than that of commercial bovine gelatin. Therefore, it presents a viable alternative to mammalian gelatins. Furthermore, microwave‐extracted gelatin demonstrated enhanced gel strength, viscosity, and thermal properties, leading to an overall improvement in the quality of chicken feet gelatin.

## Graphical abstract

The aim of this study was to investigate the physicochemical properties of chicken feet gelatin, which is extracted by acidic and microwave treatment in comparison with a commercial bovine gelatin.

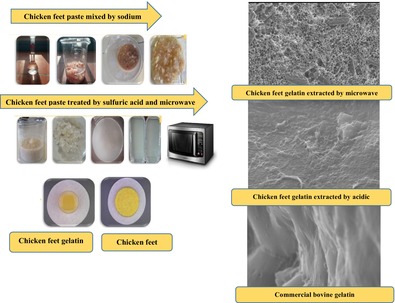

## 1 Introduction

Consumption of animal by‐products has increasingly grown over the last decade. From the past to the present, most commercial gelatin has primarily been sourced from pigs and cows. However, cultural, and religious restrictions and the recurrence of diseases such as bovine spongiform encephalopathy that affect human health have led researchers to pay attention to other gelatin sources (Abedinia et al. 2020). Some studies have investigated collagen replacement from various animal sources, such as aquatic species and poultry (Kaewdang et al. 2016; Mohtar et al. 2011; Mokrejš et al. 2019).

Currently, most research focuses on gelatin production from fish. However, the gel form of fish gelatin is less stable and exhibits poorer rheological properties compared to gelatin derived from mammals. Additionally, studies have reported allergic reactions associated with fish gelatin (Al‐Nimry et al. 2021).

Poultry processing industries produce a large number of by‐products in the form of heads, feet, bones, viscera, and feathers (Santana et al. 2020). Among these by‐products, chicken feet are a good source for gelatin production (da Almeida, da Silva Lannes, et al. 2012). Based on limited studies of chicken feet gelatin, it has been found to contain higher levels of glycine, hydroxyproline, and proline, and it exhibits greater thermal stability than gelatin derived from mammals or fish (Bichukale et al. 2018; Nik Aisyah et al. 2014). Therefore, chicken feet are used as a source for gelatin production. Gelatin is a high molecular weight polypeptide (Gál et al. 2020). During the thermal denaturation of collagen, structural changes and intramolecular bond breakage occur, along with chemical and physical changes that eventually lead to the gelatin production. The gelatin properties like melting temperature and gel strength are the most important characteristics that determine its commercial quality. The properties are influenced by some agents, like amino acid composition and molecular weight distribution (Perez‐Puyana et al. 2019).

Gelatin is extensively used in the food industry, health, pharmaceuticals, and photography products. There are various methods for extracting gelatin from animal tissue cells, and the difference in these methods depends on the sources (Al‐Hassan 2020). One of the best methods for gelatin extraction is the acidic treatment because the highest quality of gelatin is obtained through acidic treatments (Abedinia et al. 2020). Acidic treatment makes collagen swell and increases gelatin extraction efficiency during thermal hydrolysis. Further swelling and liquefaction of collagen are strongly influenced by the acid concentration (Damrongsakkul et al. 2008). Another method of gelatin extraction is microwave treatment, which is proposed as an efficient technique to enhance molecular and physicochemical properties like viscosity, gel strength, and melting point (Liu et al. 2019). So, in this research, the acidic and microwave treatments for gelatin extraction were compared in terms of the yield of extraction, gelatin strength, color, viscosity, and gelling and melting temperatures. Also, the microstructure and amino acid profile were determined.

## 2 Materials and Methods

### 2.1 Materials

Chicken feet were purchased from the protein products market in Babol, Iran. Chemicals such as sulfuric acid (98% purity), NaOH, and all other reagents used in the research were prepared by the German company Merck. Commercial bovine gelatin was also purchased from Kia Tejarat Batis Company in Iran.

### 2.2 Methods

#### 2.2.1 Extraction of Gelatin

The purchased chicken feet were packed in nylon bags and transported to the laboratory. In the laboratory, chicken feet were washed several times to remove blood and other residual materials. In the next step, the chicken feet were cut into smaller pieces and placed into a meat grinder and processed into a paste.

Gelatin extraction was performed according to Liu et al. (2019) the method with some changes. For extraction, the chicken feet paste was first washed with warm water. Then, chicken feet paste (100 g) mixed in 0.5 M of NaOH (1:2 w/v) using a stirrer (Pars Azma, Iran) at 50 rpm for 10 min to eliminate non‐collagenous proteins. In the next step, the sample was washed with cold water until its pH was neutralized and placed into the sample in a three‐layer filter to completely remove excess water. Then, acidic treatment for gelatin extraction was optimized based on the effect of sulfuric acid concentration (1–3 N), the weight of chicken feet paste to the acid solution ratio (1:2–6 w/v), temperature (60°C–90°C) and time (1–5 h). For optimal acidic gelatin extraction, a 2 N sulfuric acid solution with a 1:4 paste‐to‐acid ratio at 76.40°C for 3 h should be used to achieve maximum yield and gel strength. The pH is adjusted to 5.5–6 by washing the chicken feet paste. Then, the chicken feet paste is filtered to remove excess water. The microwave treatment was optimized based on the yield and gel strength by sulfuric acid (1–3 N), microwave power (360–720 W) and time (10–20 min). Based on the results, for optimum microwave chicken feet gelatin extraction, 2 N sulfuric acid should be applied in 540 W for 20 min to reach maximum yield and gel strength. The extracted solution was then filtered through a three‐layer filter, poured into aluminum containers, and dried at 45°C in a hot air flow oven (Mort, Germany ULM40). After the gelatin solution dried, the gelatin sheets were broken into smaller pieces and powdered by a Bosch MKM6000 grinder (Germany) and passed through a sieve. Gelatin powder was stored in a refrigerator at 4°C in polyethylene bags until used in experiments.

#### 2.2.2 Gelatin Analysis

##### 2.2.2.1 Extraction Yield

The extraction yield of gelatin was determined based on the weight ratio of gelatin powder to the weight of chicken feet paste, based on the following equation (Rafieian et al. 2015), (Equation 1).

(1) $$\text{Extraction yeild}=\frac{\mathrm{Dry}\;\text{gelatin powder}\;\left(w\right)}{\text{Chicken feet paste}\;\left(w\right)}\times 100$$

##### 2.2.2.2 Gel Strength Determination

The bloom, which is the most industrial grading parameter for gelatin, is the gel strength. Each gelatin type produces different gel strengths, resulting in variations in texture (Aidat et al. 2023). The gel strength obtained from acidic, microwave‐treated, and commercial gelatins was measured using a Brookfield texture analyzer (Ct3‐10, USA). First, a 6.67% gelatin solution was prepared, then before measuring the gel strength at 5°C–7°C, it was placed in the refrigerator for 16 h. After that, the gel strength was measured using a cylinder probe (12.7 mm diameter) at a speed of 1 mm/s with a penetration depth of 4 mm, and the bloom (gel strength) was recorded in grams (Fernandez‐Dıaz et al. 2001).

##### 2.2.2.3 Analysis of Amino Acid Profile by HPLC

The amino acid profile was analyzed by high performance liquid chromatography (HPLC) (Infinity Isocratic LC 1220, USA). Gelatin powder (15 mg) was digested with 6 M of hydrochloric acid for 20 h at 110°C. Then, a small amount of hydrolyzed gelatin was mixed with 0.5 mL of α‐aminobutyric acid, followed by the addition of 100 mL deionized water to the mixture. The resulting solution was filtered and injected into the HPLC (Mirzapour‐Kouhdasht et al. 2019).

##### 2.2.2.4 FT‐IR Spectroscopy

FT‐IR spectroscopy is a non‐destructive and rapid method to determine changes in protein bonds and the secondary structure of proteins during the collagen to gelatin conversion (Santana et al. 2020). The FT‐IR spectrum of gelatin samples was recorded using an infrared spectrometer (Technologies 630, Cary 2017, Agilent, USA). Gelatin samples (4 mg) were analyzed in the mid‐infrared region (1400–4000 cm^−1^) at 25°C. Spectral data were analyzed using specialized software (Sadat Hosseini et al. 2025).

##### 2.2.2.5 Differential Scanning Calorimetry (DSC)

The thermal properties of gelatin were investigated using a DSC‐500 (Sanaf, Iran). Gelatin powder (15 mg) was scanned from 30°C to 90°C with a heating rate of 10°C/min and its melting temperature was calculated (Sarbon et al. 2015).

##### 2.2.2.6 Gelling and Melting Temperature Analysis

Gelling and melting temperature are crucial properties of gelatin quality. These factors influence the gelatin applications in the food industry. Gelatin samples (6.67 g/100 mL) were prepared using a rheometer (MCR‐301, Anton Parr, Austria) equipped with a steel plate and thermal circulator to control the temperature using a cone and plate probe (Farahmandfar et al. 2019). Measurements were performed by a temperature sweep from 50°C to 5°C with a constant strain of 1°C. Frequency stress and oscillation were adjusted to 1 Hz and 3 Pa, respectively. The loss modulus (G″), storage modulus (G′) and phase angle (δ) were measured during the cooling and heating phases (Cao et al. 2020).

##### 2.2.2.7 Colorimetric Analysis

Transparency and color are essential properties of gelatin quality. The color parameters (L*, a*, b*) of gelatin powder were measured using a colorimeter (IMG Pardazesh CAM‐SystemXI). L* shows lightness to darkness, a* green to red, and b* blue to yellow spectrum (Widyasari and Rawdkuen 2014). The other parameters such as ∆E and BI, which are calculated by Equations (2) and (3). ∆E indicates the color difference (Saenmuang et al. 2020) and BI is the browning index, which indicates the degree of color change to brown (Kahyaoglu 2008), (Equations (2), (3)).

(2) $$∆E=\sqrt{∆L^{+2}+∆a^{+2}+∆b^{+2}}$$

(3) $$\mathrm{BI}=100\times \frac{X-0.31}{0.17}$$

##### 2.2.2.8 Viscosity Measurement

Gelatin viscosity was measured using a Brookfield DVII Ultra viscometer (Brookfield, USA). A 6.67% gelatin solution was prepared and measured by spindle LV‐SC4‐18 at 100 rpm at 40°C (Ratnasari 2016).

##### 2.2.2.9 Turbidity Determination

Gelatin powder was mixed with water at 60°C to reach 6.67 g/100 mL. The mixture was stirred until it was thoroughly dissolved. Finally, the gelatin turbidity was evaluated at 660 nm using an Alpha‐1502 UV/Vis spectrophotometer at room temperature (25°C) (Yasin et al. 2017).

##### 2.2.2.10 Microstructures of Gelatins

Gelatin gels (6.67 g/100 mL and 2–3 mm thickness) were fixed with 2.5 g/100 mL glutaraldehyde in phosphate buffer (0.2 M, pH 7.2) for 12 h at 4°C and then washed in phosphate buffer. Then, the gels were dehydrated using different ethanol solutions. The gels were mounted on a bronze stub and sputter‐coated with gold (Ion‐Coater, KIC‐1COXEM). Microstructure of extracted gelatin gel was visualized by scanning electron microscopy (SEM) (JEOL‐JSM‐6390 LV, Tokyo, Japan) at 20 kV (Kittiphattanabawon et al. 2010).

### 2.3 Statistical Analysis

The experiments were done in triplicate (*n* = 3), except for the amino acid profile (*n* = 1) and the rheological data (*n* = 2). Data are shown as means±SD and analyzed by two‐way ANOVA, followed by the mean comparisons by *t*‐test. Analyses were done to a significance level of *p* < 0.05, using SPSS 17.0 for Windows (SPSS Inc., Chicago, IL, USA).

## 3 Results and Discussion

### 3.1 Yield Extraction of Chicken Feet Gelatin

In this study, the yield of acidic gelatin extraction was 11.44% compared to 10.48% in microwave extraction. The yield of gelatin extraction not only depends on the gelatin source but also the pretreatment process acts as a vital role in its efficiency. Cheng et al. (2009) used four acids as a pretreatment for chicken feet gelatin extraction. The results showed that in all treatments, the extraction yield of gelatin using lactic acid (8.34%) and acetic acid (7.31%) was higher than HCl (5.66%). According to Lee et al. (2012), chicken collagen is heavily digested with HCl during pretreatment. A low yield in gelatin extraction from chicken feet was also confirmed by (deAlmeida, deAraújo, and Santana 2012). Moreover, Sarbon et al. (2015) extracted gelatin from chicken skin using an alkaline followed by acid treatment and the yield was 16.2%.

### 3.2 Gel Strength of Chicken Feet Gelatin

The gel strength of acidic‐extracted and microwave‐extracted gelatin was 206 g and 256 g, respectively. Meanwhile, the gel strength of the bovine gelatin was 200 g. The gel strength strongly depends on α‐chain of gelatin. The high proportion of peptides with higher or lower molecular weight than α‐chains can reduce the gel strength. Therefore, gelatin containing more α‐chains exhibits greater gel strength. The stability of the triple helical structure in renatured gelatins is proportional to the pyrrolidine imino acid content. Although proline is important, hydroxyproline is believed to contribute significantly to stabilizing the triple collagen helix (Gómez‐Guillén et al. 2011).

Aykın‐Dinçer et al. (2017) reported the gel strength of broiler skin gelatin is 166 g. It was also found that chicken skin and tendon gelatin at 3.33% had a lower gel strength than gelatin at 6.67%. Therefore, the gel strength increases with the gelatin concentration. The collagen gel strength results extracted from chicken feet using acids such as acetic, lactic, and citric acids in different concentrations showed the gelatin gel strength varied from 123 to 204 g. Gelatin extracted using 1.5% (v/v) acetic acid showed the highest bloom (204 g) (Chakka et al. 2017). In the research of Liu et al. (2019) that extracted rabbit skin gelatin using microwave treatment, the gel strength exceeded 400 g. The gelatins extracted via microwave for 5, 15, and 30 min showed significantly higher gel strength compared to water bath extraction, indicating that the shorter microwave times lead to improved gel strength. Indeed, microwave extraction can break down its subunits, resulting in higher gel strength. However, as the microwave treatment duration increased from 60 to 90 min, gel strength decreased significantly (*p* < 0.05). This decline may be due to a reduction in amino acid content or the degradation of high molecular weight subunits. The research showed the microwave extraction showed the highest gel strength among the other extraction methods (*p* < 0.05). A short microwave extraction time may allow collagen extraction to occur before complete protein denaturation, explaining the reported gelatin gel strength of 260 g via microwave extraction, which was comparable to this study (Park et al. 2013). Moreover, the gel strength of gelatin extracted from duck skin using water bath and microwave methods was higher than that obtained through other techniques (Park et al. 2013).

Feng et al. (2021) investigated microwave irradiation as a new approach to improving the physicochemical properties of pig skin gelatin. It was found that with increasing the time of microwave irradiation, the gel strength gradually decreased.

### 3.3 Amino Acid Analysis of Chicken Feet Gelatin

The amino acid analysis of chicken feet gelatin in two acidic and microwave extraction methods compared to commercial bovine gelatin is shown in Table 1. The most abundant amino acids in commercial gelatin and chicken feet were glycine and proline, and the lowest amino acids were tyrosine, isoleucine, and histidine. Amino acids, especially proline and hydroxyproline, create the collagen structure's strength, and their relatively limited content leads to the formation of helices with less steric hindrance and effect on the gelatin's dynamic properties (Amiza and Siti Aishah 2011).

**TABLE 1:** Amino acid composition of gelatin extracted from chicken feet and commercial bovine gelatin.

Amino acid	Gelatin extracted by microwave treatment (%)	Gelatin extracted by acidic treatment (%)	Bovine gelatin (%)
ASP	4.07	3.75	4.55
SER	5.20	4.72	5.03
GLU	7.89	8.13	7.76
GLY	21.74	21.61	21.02
HIS	0.43	0.43	0.63
ARG	6.43	6.69	7.01
THR	3.36	2.70	3.15
PRO	10.66	10.76	10.11
ALA	6.70	5.83	6.80
TYR	0.60	0.53	0.47
VAL	2.33	0.76	2.20
MET	2.60	2.42	2.72
LYS	4.45	4.07	3.82
ILEU	0.57	0.43	0.62
LEU	2.70	2.90	3.18
PHE	2.60	2.66	2.66

The researchers also reported that the gelatin is related to the total amino acid and glycine. Changes in the amino acids like alanine, glycine, and amino acids affect functional properties such as foaming and emulsifying properties (Xu et al. 2011). The study suggested the amino acid profiles of chicken head and turkey gelatins had similar glycine, proline, and hydroxyproline content, which is approximately 32%, 12%, and 11% of the total amino acids, respectively. All poultry head gelatins had very low tyrosine content and were free of histidine. In this study, tryptophan and cysteine were not detected. Tryptophan and cysteine may be entirely eliminated by acid hydrolysis during amino acid analysis (Du et al. 2013).

As it is shown in Table 1, the chicken feet gelatin extracted by acidic and microwave methods, as well as commercial bovine gelatin, had low histidine content The research on the characterization of gelatin from black‐bone chicken by‐products resulted in the quantification of 19 amino acids. High glycine, arginine, and hydroxyproline content, as well as low methionine, cystine, and tryptophan content, were detected. The study also found that the amino acid content of chicken bone gelatin was significantly higher than that of commercial gelatin (Saenmuang et al. 2020). The essential amino acids in this study were higher than those in the gelatin from black‐bone chicken research.

### 3.4 FTIR Analysis of Chicken Feet Gelatin

**FIGURE 1:**
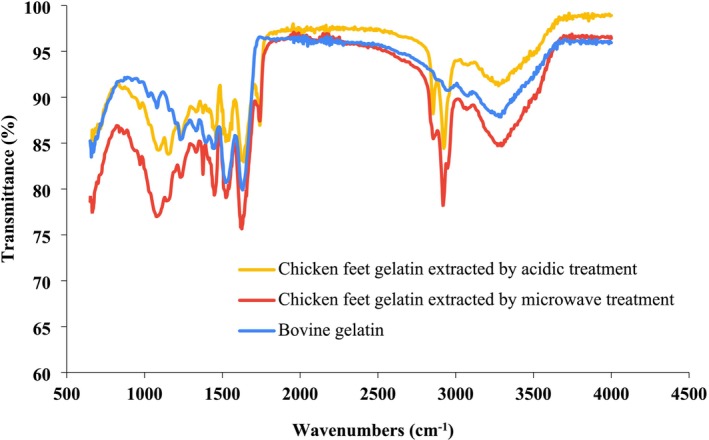
FTIR spectra of chicken feet gelatin extracted by acidic and microwave and bovine gelatin.

Infrared (IR) spectroscopy is one of the most potent spectroscopic techniques for food analysis. It detects the details of the functional groups and the chemical compounds. The Fourier transform infrared (FTIR) technique can evaluate a product's shelf life (Saenmuang et al. 2020). IR spectroscopy is applied to determine the secondary structure of proteins, monitor structural changes and dynamics, and assess stability. The FTIR of chicken feet gelatin and bovine gelatin was analyzed. The FTIR spectrum of gelatin samples was recorded in the range of 400–4000 (cm^−1^) at ambient temperature (Figure 1). The FTIR spectrum of chicken feet gelatin extracted by optimized acidic and microwave treatment showed that the main peaks contained amide groups (I, II, III, A, and B), which are characteristic of proteins. While in commercial bovine gelatin samples, the B band was not observed. Therefore, producing gelatin from chicken feet is an effective way to utilize poultry industry waste. Additionally, chicken gelatin has better nutritional quality than commercial bovine gelatin.

The infrared spectra of proteins are characterized by peaks corresponding to amide groups. The most prominent spectrum in the identification of the proteins' secondary structure is the amide I band, which usually appears in the range of 1600–1700 cm^−1^. The amide I band probes stretching vibration of the C=O bonds in the peptide backbone, which is very helpful in the analysis of the proteins' secondary structure (Hashim et al. 2014). The stretching frequency depends on the unique molecular geometry and the hydrogen bonds of the C=O and N‐H groups, particularly relevant to the proteins' secondary structure. Amide II derives from in‐plane N‐H bending (40%–60% of the potential energy) and C‐N stretching (18%–40%), leading to less specificity and sensitivity for conformational protein changes. Amide III shows a combination of C‐N stretching vibration and N‐H deformation, as well as vibration‐induced adsorption of CH_2_ groups of the proline side chain and the glycine backbone. The amide A indicates the involvement of N‐H groups of shorter peptide fragments in the hydrogen bond, as well as a tendency to join with the CH_2_ stretch peak. Amide B shows the interaction of the ‐NH_3_ group between peptide chains (Kaewdang et al. 2016). Therefore, the secondary structure of chicken feet gelatins has been affected by acid pretreatment and microwave.

Table 2 shows the values of different spectra (FTIR) of the optimal sample of chicken feet gelatin extracted using acidic and microwave treatments, as well as commercial bovine gelatin. The lower the intensity of amide I, II, and III shows irregularity in the gelatin structure and the lack of a triple helix (Muyonga et al. 2004). The optimal microwave gelatin extraction has a more regular structure than the acidic‐extracted gelatin and the commercial bovine sample. The FTIR spectrum of chicken feet collagen was investigated by Santana et al. (2020). ATR‐FTIR analysis showed that 70.90% of the total protein in chicken feet is collagen, whereas in commercial bovine gelatin, only 30.31% is collagen. The study suggested chicken feet gelatin using acidic, alkaline, and enzymatic processes. Chicken feet collagens FTIR spectra showed that absorption peaks include amides A and B, I, II, and III. Therefore, chicken feet collagen was present in both extraction methods (Zhou et al. 2016).

**TABLE 2:** FTIR spectra of chicken feet gelatin extracted by acidic and microwave treatments compared by bovine gelatin.

Amide	Gelatin extracted by microwave (cm^−1^)	Gelatin extracted by acidic treatment (cm^−1^)	Bovine gelatin (cm^−1^)
Amide A	3271.348	3277.132	3212.609
Amide B	2949.728	2854.444–2924.685	0
Amide I	1625.379–17470.951	1628.383–1741.300	1631.726
Amide II	1528.331	1541.295	1523.695
Amide III	1234.947–1375.795	1376.278	1236.424

### 3.5 DSC Analysis of Chicken Feet Gelatin

Differential scanning calorimetry (DSC) is a prominent tool for investigating the gelatin thermal properties. It is a method used to evaluate temperatures and enthalpies related to phase transitions, and it has found widespread application in the food industry. DSC is usually used to test crystal structures and determine the melting temperature. The biological materials in their amorphous state (thermally and chemically) are less stable than crystalline structures. As the heat capacity increases with temperature, the DSC curve shows a slight upward slope toward a higher temperature (Castro et al. 2022; Mukherjee and Rosolen 2013).

**FIGURE 2:**
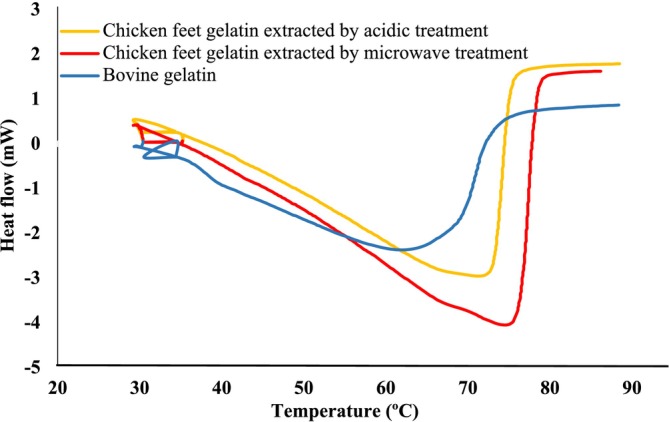
Differential scanning calorimetry thermograph of chicken feet gelatin extracted by acidic and microwave treatment and commercial bovine gelatin from 30°C to 90°C.

DSC analysis of chicken feet gelatin and commercial bovine gelatin is shown in Figure 2. The melting temperature of chicken feet gelatin in both microwave and acidic treatment was higher than that of commercial gelatin. The lower melting temperature shows that the structural stability of the commercial gelatin is weaker than that of chicken feet gelatin, and gelatin extracted by microwave is stronger than that of acidic gelatin treatment. Also, chicken gelatin has a higher enthalpy, which is attributed to a more stable collagen structure. Benjakul et al. (2010) claimed that higher thermal conductivity (DSC) and higher enthalpy change (AH) during cooling and heating indicated that chicken gelatin was more stable against heat, probably due to high proline and hydroxyproline.

### 3.6 Microstructure of Chicken Feet Gelatin

**FIGURE 3:**
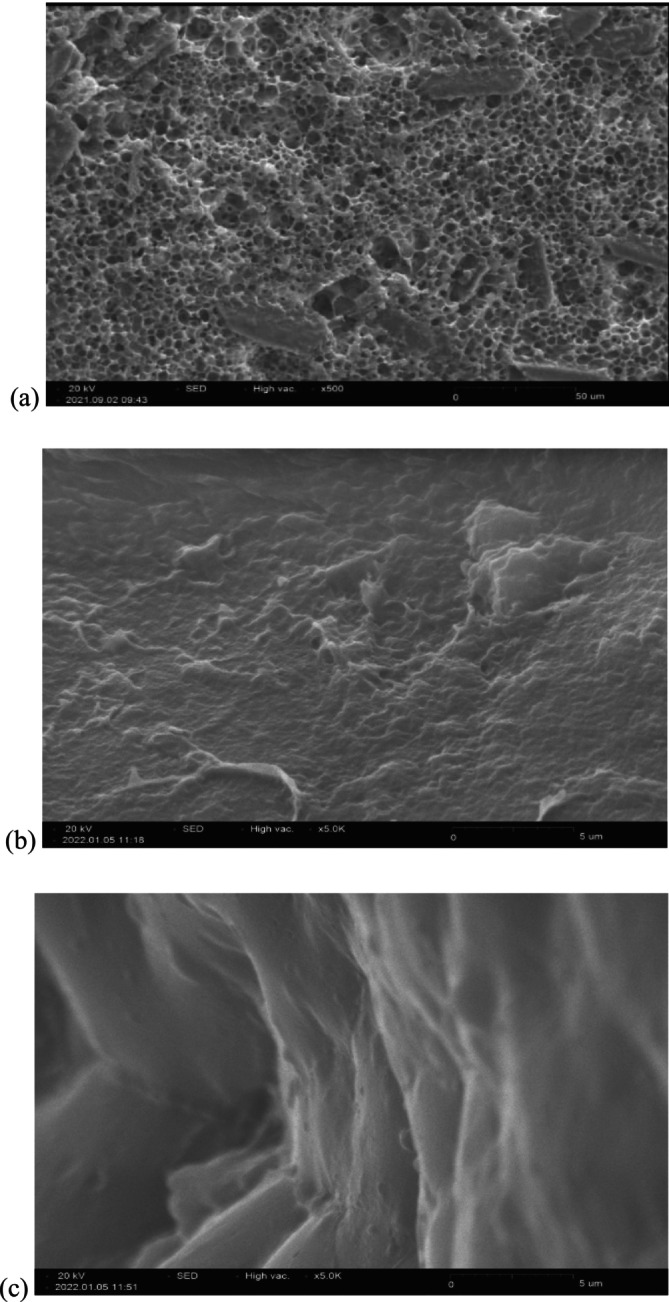
Images of scanning electron microscopy (SEM) of gelatin samples: (a) chicken feet gelatin extracted by microwave, (b) gelatin extracted by acidic treatment and (c) bovine gelatin.

A scanning electron microscope (SEM) is an electron microscope for analyzing the morphology of food nanostructures. The SEM results of chicken feet gelatin extracted by acidic and microwave treatment and commercial bovine gelatin are presented in Figure 3 with 5000× magnification. Figure 3a is chicken feet gelatin extracted by microwave (gel strength 253 g). This sample shows a uniform structure with small holes and regular cross‐links between protein segments. The high strength and viscosity properties of the gel result in a spongy structure. The uniform structure of small cracks in the gelatin matrix is attributed to its higher water absorption capacity. Figure 3b shows chicken feet gelatin extracted by acidic treatment with a lower gel strength (206 g) and a non‐uniform structure without holes. Figure 3c shows commercial bovine gelatin showing a surface without holes like acidic chicken feet gelatin. At low temperature, the gelatin surface remains relatively smooth. In the acidic extraction, the intermolecular cross‐links of the collagen triple helix structure are hydrolyzed and convert collagen to gelatin.

The agitation of water molecules and charged ions is the primary mechanism of microwave heating, causing gelatin to heat up instantly. However, the morphology of gelatin extracted using microwave treatment remains intact after 20 min (Liu et al. 2019). The study analyzed the microstructure of chicken and turkey gelatin. The results showed the surface with multiple small and uniform pores, which indicated significant gel properties (Du et al. 2013). Shahiri Tabarestani et al. (2016) showed the fish skin gelatin structure extracted by acidic and alkaline treatments. The findings revealed that acidic gelatin exhibited heterogeneous and irregular structures and that the gelatin network was influenced by the pretreatment conditions.

### 3.7 Viscosity Analysis of Chicken Feet Gelatin

Molecular weight and distribution of gelatin polypeptides are two important factors of viscosity. Viscosity is also more significantly influenced by measurement methods, raw material properties, and gelatin extraction methods (Asnaashari et al. 2016; Tümerkan et al. 2019). It has also been claimed that the molecular weight distribution has a more significant effect on viscosity than the gelatin amino acid composition (Mokrejš et al. 2019). The lower gelatin viscosity may be due to the low molecular weight peptide chains and excessive collagen hydrolysis in the pretreatment. Low viscosity gelatin produces a brittle and weak gel, while high viscosity gelatin produces a hard and flexible gel that has a more value. Therefore, high viscosity gelatins are preferred in the food industry (Norziah et al. 2014).

The results of chicken feet gelatin viscosity are shown in Table 3. The highest viscosity is attributed to chicken feet gelatin extracted by microwave (24.18 cp), followed by chicken feet gelatin extracted by acidic treatment (8.47 cp) and commercial bovine gelatin (5.46 cp) (*p* < 0.05). Due to the low extraction time in the microwave, the viscosity of the gelatin has increased. More prolonged extraction has a negative effect on viscosity. Also, the viscosity is related to the gel strength. The study reported the viscosity of chicken bone gelatin was 5.85 cp (Rafieian et al. 2015). Ahmad et al. (2019) used two plant enzymes called actinidine and papain to pretreat bovine skin at optimum condition. The viscosity results were from 12.10 to 13.10 cp, which was less than the gelatin extracted by microwave in this study. The gelatin viscosity of chicken head extracted by proteolytic enzyme was in the range of 1.4 to 9.5 mp/s. The results suggested that higher enzyme concentration and longer extraction times negatively affected the gelatin viscosity (Gál et al. 2020). Moreover, the viscosity of chicken skin gelatin changed from 6.1 to 7 cp under the influence of extraction temperature (Tümerkan et al. 2019).

**TABLE 3:** Viscosity of optimal samples of chicken feet gelatin and commercial bovine gelatin.

Sample	Viscosity (cp)
Acidic chicken feet gelatin extraction	8.47 ± 0.07^b^
Microwave chicken feet gelatin extraction	24.18 ± 0.02^c^
Bovine gelatin	5.46 ± 0.04^a^

*Note:* Different letters in the column indicate significant differences (*p* < 0.05).

### 3.8 Melting and Gelling Temperature of Chicken Feet Gelatin

**FIGURE 4:**
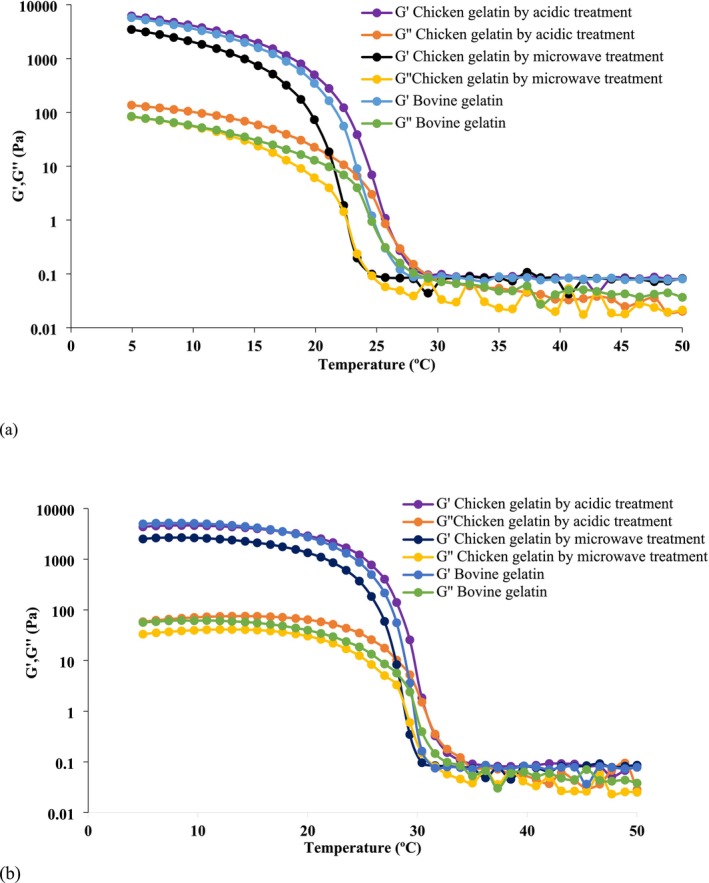
Viscoelastic properties (G′ and G″) of 6.67% of bovine and chicken feet on (a) cooling from 50°C to 5°C and (b) heating process from 5°C to 50°C.

The G″ and G′ of gelling solutions as a function of temperature during (a) cooling (50°C to 5°C) and (b) heating (5°C to 50°C) are shown in Figure 4. During the cooling, G′ and G″ increase sharply, but in the heating the G′ and G″ decrease. The G′ was higher than G″ in both the heating and cooling. The melting and gelling temperatures of the gelatin samples are shown in Table 4. The highest melting temperature is related to the chicken feet gelatin extracted by acidic treatment (30.76°C) (*p* < 0.05) and the lowest melting temperature was related to the chicken feet gelatin extracted by microwave treatment (28.85°C), which it has the lowest gel formation temperature (22.7°C). Also, the Mirzapour‐Kouhdasht et al. (2019) study suggested melting (19.61°C) and gelling temperature (3.67°C) of carp by‐product gelatin in the microwave method lower than melting (22.85°C) and gelling (6.67°C) temperature in ultrasound method.

**TABLE 4:** Melting and gelling temperature of chicken feet gelatin compared with commercial bovine gelatin.

Sample	Melting point (°C)	Gelling point (°C)
Acidic extraction	30.76 ± 0.34^a^	26.65 ± 1.05^a^
Microwave extraction	28.85 ± 0.35^c^	22.7 ± 0.5^b^
Bovine gelatin	29.65 ± 0.15^b^	23.35 ± 1.05^a^

*Note:* Different letters in the column indicate significant differences (*p* < 0.05).

According to the findings of Gokhan Boran and Regenstein (2010), commercial gelatin from chicken, pig, and bovine skin had a high melting temperature of more than 30°C. In the present study, except for the acidic extracted sample (30.76°C), the rest of the samples have lower melting point temperatures. The acidic extracted gelatin had a significantly different melting temperature (30.76°C) and higher gel formation temperature (26.65°C) (*p* < 0.05). So, it had more bonds that formed the firm gel network. High melting temperature indicates stronger interactions in the gel system and higher thermal stability. Gel formation is affected by pH, concentration, relative composition of α‐, β‐ and γ‐chain components, molecular weight, and temperature (Gómez‐Guillén et al. 2011). The G Boran et al. (2010) research suggested that the difference between melting and gelation temperatures was 7°C for commercial chicken, pig, and bovine gelatin samples, but 10°C for fish skin gelatins. The difference between the melting and gel formation temperature of chicken feet gelatin extracted by acidic treatment and bovine gelatin was 3.2°C, but for the gelatin extracted by microwave treatment, it was 4.25°C. In fact, this feature makes chicken feet gelatin unique to use in the food industry.

### 3.9 Colorimetric Analysis of Chicken Feet Gelatin

Gelatin color is significantly affected by gelatin source and extraction, which affects consumer acceptance (Nuñez et al. 2023). According to Bichukale et al. (2018), more pretreatment time leads to non‐enzymatic browning and reduced L*. However, gelatin color does not affect its functional properties. The drying method also affects the gelatin color (Shyni et al. 2014). Table 5 shows the color values of gelatin samples. The highest L* that indicates the brightness belonged to the microwave. Also, a significant difference was observed between the chicken feet gelatin extracted by the optimal microwave method and the commercial sample (*p* < 0.05). Bovine gelatin was shown to have the highest ∆E compared to chicken feet gelatin. Also, the browning index of gelatin extracted by acidic treatment was more than other treatments. Non‐enzymatic browning reactions occurred between the free amine groups of protein and the hydroxyl glucoside group of reducing sugars or carbonyl compounds such as aldehydes and ketones during the process. As the amino and hydroxyl compounds increase, the rate of non‐enzymatic browning reactions also enhances (Nath et al. 2022).

**TABLE 5:** Color parameters of chicken feet and commercial gelatins.

Gelatin	L*	a*	b*	∆E	BI
Acidic extraction	54.78 ± 1.65^b^	−2.85 ± 0.13^b^	26.0 ± 0.74^a^	45.65 ± 1.78^b^	52.94 ± 2.31^a^
Microwave extraction	66.27 ± 0.51^a^	−4.78 ± 0.39^c^	21.65 ± 0.81^b^	34.0 ± 0.45^c^	41.29 ± 1.92^b^
Bovine	8.51 ± 1.87^c^	−0.59 ± 0.12^a^	2.83 ± 0.56^c^	84.01 ± 1.85^a^	35.29 ± 1.70^c^

*Note:* Different letters in the column indicate significant differences (*p* < 0.05).

The research investigated two gelatins extracted from chicken feet and heads; it indicated the color factors of the chicken feet gelatin (L* = 17.93, a* = 1.7, b* = 53) and chicken head gelatin (L* = 15.30, a* = 1.73, b* = 62) were similar to the present study (Rafieian et al. 2015). In another study, researchers investigated different gelatin extraction methods from duck skin and showed the highest L* was 42.56 for ultrasonic gelatin extraction, followed by microwave extraction (L* = 28). Meanwhile, the lowest L* was for the superheated steam extraction (Kim et al. 2020). Park et al. (2013) reported the color parameters of microwave gelatin extracted from duck feet. The results of color parameters were L* = 1.19, a* = 0.99, and b* = 34, respectively.

### 3.10 Turbidity of Chicken Feet Gelatin

As shown in Table 6, the turbidity of the chicken feet gelatin sample extracted by acidic and microwave treatment is 1.62 and 2.47 ppm, respectively, which is higher than the turbidity of commercial bovine gelatin, 0.94 ppm (*p* < 0.05). The gelatin containing more polymer masses with high molecular weight leads to high turbidity, and turbidity changes could be due to a slight reduction in the number of particles and size or refractive index changes. Gelatin extraction at higher temperatures leads to random accumulation of protein molecules, leading to increased turbidity (Montero and Acosta 2020) Gelatin turbidity is usually caused by minerals and proteins that are not removed during extraction (Du et al. 2013). The results of fish skin gelatin extracted with different acid treatments showed that the propionic acid treatment had the highest turbidity (1.5 ppm) and citric acid treatment had the lowest turbidity (0.05 ppm) (Gómez‐guillœn and Montero 2001). In the study of the goat skin gelatin by freeze and spray drying and compared it with commercial bovine gelatin, the turbidity of freeze‐dried gelatin was 1.1 ppm, while spray‐dried gelatin had a turbidity of 1.2 ppm (Mad‐Ali et al. 2017).

**TABLE 6:** Optimal sample turbidity of chicken feet gelatin and commercial bovine gelatin.

Sample	Turbidity (ppm)
Acidic chicken feet gelatin extraction	1.62 ± 0.01^b^
Microwave chicken feet gelatin extraction	2.74 ± 0.04^a^
Bovine gelatin	0.94 ± 0.03^c^

*Note:* Different letters in the column indicate significant differences (*p* < 0.05).

## 4 Conclusion

Most animal by‐products contain a large amount of collagen‐rich connective tissue which can be applied for various applications in the pharmaceutical and food industries. Collagen has been extracted from pigs and cattle and converted into mammalian gelatin. However, due to health and safety issues like bovine spongiform encephalopathy related to bovine gelatin, and cultural‐religious issues related to pig gelatin consumption, new collagen sources were proposed based on consumer demand. Poultry gelatin may be a valuable alternative to common gelatin. In this research, the possibility of high‐quality gelatin production from poultry by‐products was researched. Chicken feet gelatin is classified as a high‐bloom. High gel strength gelatin has higher water absorption and melting temperature than low gel strength ones and can be used in dairy products, desserts, and jelly‐based meats.

In the optimized acidic treatment, gelatin has a maximum yield (11.99%) and gel strength (206 g). Also, the optimized condition (540 W for 20 min) of microwave gelatin extraction leads to 253 g gel strength. The viscosity of chicken feet gelatin was higher than that of commercial bovine gelatin. Analysis (FTIR) showed all the amide bands in acidic gelatin and microwave, but not in commercial gelatin (band B). In temperature scanning and gel formation analysis, the highest melting temperature and gel formation were related to gelatin extracted by acidic treatment. The microscopic structure of the gelatin extracted by microwave treatment compared to the other two samples had numerous holes and showed the highest turbidity. Therefore, microwave‐extracted gelatin could serve as a viable substitute for mammalian gelatin. Compared to acidic‐extracted gelatin, microwave‐extracted gelatin demonstrated higher gel strength, viscosity, and thermal properties, enhancing the overall quality of chicken feet gelatin.

## Author Contributions

**Hanieh Esmaeili‐Kaliji:** data curation (equal), formal analysis (equal), investigation (equal), resources (equal), software (equal). **Reza Farahmandfar:** conceptualization (equal), methodology (equal), project administration (equal), supervision (equal), writing – review and editing (equal). **Ali Motamedzadegan:** methodology (equal), resources (equal). **Maryam Asnaashari:** conceptualization (equal), methodology (equal), validation (equal), writing – original draft (equal), writing – review and editing (equal).

## Conflicts of Interest

The authors declare no conflicts of interest.

## Data Availability

The data that support the findings of this study are available on request from the corresponding author. The data are not publicly available due to privacy or ethical restriction.

## References

[fsn370651-bib-0001] Abedinia, A. , A. M. Nafchi , M. Sharifi , et al. 2020. “Poultry Gelatin: Characteristics, Developments, Challenges, and Future Outlooks as a Sustainable Alternative for Mammalian Gelatin.” Trends in Food Science & Technology 104: 14–26.

[fsn370651-bib-0002] Ahmad, T. , A. Ismail , S. A. Ahmad , et al. 2019. “Physicochemical Characteristics and Molecular Structures of Gelatin Extracted From Bovine Skin: Effects of Actinidin and Papain Enzymes Pretreatment.” International Journal of Food Properties 22, no. 1: 138–153.

[fsn370651-bib-0003] Aidat, O. , L. Belkacemi , M. Belalia , M. khairi Zainol , and H. S. Barhoum . 2023. “Physicochemical, Rheological, and Textural Properties of Gelatin Extracted From Chicken By‐Products (Feet‐Heads) Blend and Application.” International Journal of Gastronomy and Food Science 32: 100708.

[fsn370651-bib-0004] Al‐Hassan, A. A. 2020. “Gelatin From Camel Skins: Extraction and Characterizations.” Food Hydrocolloids 101: 105457.

[fsn370651-bib-0005] Al‐Nimry, S. , A. A. Dayah , I. Hasan , and R. Daghmash . 2021. “Cosmetic, Biomedical and Pharmaceutical Applications of Fish Gelatin/Hydrolysates.” Marine Drugs 19, no. 3: 145. 33800149 10.3390/md19030145 PMC8000627

[fsn370651-bib-0006] Amiza, M. , and D. Siti Aishah . 2011. “Effect of Drying and Freezing of Cobia (*Rachycentron canadum*) Skin on Its Gelatin Properties.” International Food Research Journal 18, no. 1: 159–166.

[fsn370651-bib-0007] Asnaashari, M. , A. Motamedzadegan , R. Farahmandfar , and T. K. Rad . 2016. “Effect of S. Macrosiphon and *L. perfoliatum* Seed Gums on Rheological Characterization of Bitter Orange ( *Citrus aurantium* L.) and Pomegranate ( *Punica granatum* L.) Paste Blends.” Journal of Food Science and Technology 53, no. 2: 1285–1293. 10.1007/s13197-015-2069-8. 27162409 PMC4837719

[fsn370651-bib-0008] Aykın‐Dinçer, E. , A. Koç , and M. Erbaş . 2017. “Extraction and Physicochemical Characterization of Broiler ( *Gallus gallus* Domesticus) Skin Gelatin Compared to Commercial Bovine Gelatin.” Poultry Science 96, no. 11: 4124–4131. 10.3382/ps/pex237 29050430

[fsn370651-bib-0009] Benjakul, S. , Y. Thiansilakul , W. Visessanguan , et al. 2010. “Extraction and Characterisation of Pepsin‐Solubilised Collagens From the Skin of Bigeye Snapper (Priacanthus Tayenus and *Priacanthus macracanthus*).” Journal of the Science of Food and Agriculture 90, no. 1: 132–138. 20355023 10.1002/jsfa.3795

[fsn370651-bib-0010] Bichukale, A. , J. Koli , A. Sonavane , V. Vishwasrao , K. Pujari , and P. Shingare . 2018. “Functional Properties of Gelatin Extracted From Poultry Skin and Bone Waste.” International Journal of Pure and Applied Bioscience 6, no. 4: 87–101.

[fsn370651-bib-0011] Boran, G. , S. Mulvaney , and J. Regenstein . 2010. “Rheological Properties of Gelatin From Silver Carp Skin Compared to Commercially Available Gelatins From Different Sources.” Journal of Food Science 75, no. 8: E565–E571. 21535497 10.1111/j.1750-3841.2010.01543.x

[fsn370651-bib-0012] Boran, G. , and J. M. Regenstein . 2010. “Fish Gelatin.” Advances in Food and Nutrition Research 60: 119–143. 20691955 10.1016/S1043-4526(10)60005-8

[fsn370651-bib-0013] Cao, S. , Y. Wang , L. Xing , W. Zhang , and G. Zhou . 2020. “Structure and Physical Properties of Gelatin From Bovine Bone Collagen Influenced by Acid Pretreatment and Pepsin.” Food and Bioproducts Processing 121: 213–223.

[fsn370651-bib-0014] Castro, J. I. , D. P. Navia‐Porras , J. A. Arbeláez Cortés , J. H. Mina Hernández , and C. D. Grande‐Tovar . 2022. “Synthesis, Characterization, and Optimization Studies of Starch/Chicken Gelatin Composites for Food‐Packaging Applications.” Molecules 27, no. 7: 2264. 35408663 10.3390/molecules27072264 PMC9000547

[fsn370651-bib-0015] Chakka, A. K. , A. Muhammed , P. Sakhare , and N. Bhaskar . 2017. “Poultry Processing Waste as an Alternative Source for Mammalian Gelatin: Extraction and Characterization of Gelatin From Chicken Feet Using Food Grade Acids.” Waste and Biomass Valorization 8: 2583–2593.

[fsn370651-bib-0016] Cheng, F.‐Y. , F.‐W. Hsu , H.‐S. Chang , L.‐C. Lin , and R. Sakata . 2009. “Effect of Different Acids on the Extraction of Pepsin‐Solubilised Collagen Containing Melanin From Silky Fowl Feet.” Food Chemistry 113, no. 2: 563–567.

[fsn370651-bib-0017] da Almeida, P. F. , S. C. da Silva Lannes , F. A. Calarge , T. M. da Brito Farias , and J. C. C. Santana . 2012. “FTIR Characterization of Gelatin From Chicken Feet.” Journal of Chemistry and Chemical Engineering 6, no. 11: 1029.

[fsn370651-bib-0018] Damrongsakkul, S. , K. Ratanathammapan , K. Komolpis , and W. Tanthapanichakoon . 2008. “Enzymatic Hydrolysis of Rawhide Using Papain and Neutrase.” Journal of Industrial and Engineering Chemistry 14, no. 2: 202–206.

[fsn370651-bib-0019] de Almeida, P. F. , M. G. O. de Araújo , and J. C. C. Santana . 2012. “Collagen Extraction From Chicken Feet for Jelly Production.” Acta Scientiarum. Technology 34, no. 3: 345–351.

[fsn370651-bib-0020] Du, L. , Z. Khiari , Z. Pietrasik , and M. Betti . 2013. “Physicochemical and Functional Properties of Gelatins Extracted From Turkey and Chicken Heads.” Poultry Science 92, no. 9: 2463–2474. 10.3382/ps.2013-03161 23960131

[fsn370651-bib-0021] Farahmandfar, R. , M. R. Salahi , and M. Asnaashari . 2019. “Flow Behavior, Thixotropy, and Dynamic Viscoelasticity of Ethanolic Purified Basil (Ocimum Bacilicum L.) Seed Gum Solutions During Thermal Treatment.” Food Science & Nutrition 7, no. 5: 1623–1633. 31139375 10.1002/fsn3.992 PMC6526669

[fsn370651-bib-0022] Feng, X. , H. Dai , L. Ma , et al. 2021. “Effect of Microwave Extraction Temperature on the Chemical Structure and Oil‐Water Interface Properties of Fish Skin Gelatin.” Innovative Food Science & Emerging Technologies 74: 102835.

[fsn370651-bib-0023] Fernandez‐Dıaz, M. , P. Montero , and M. Gomez‐Guillen . 2001. “Gel Properties of Collagens From Skins of Cod (*Gadus morhua*) and Hake (*Merluccius merluccius*) and Their Modification by the Coenhancers Magnesium Sulphate, Glycerol and Transglutaminase.” Food Chemistry 74, no. 2: 161–167.

[fsn370651-bib-0024] Gál, R. , P. Mokrejš , P. Mrázek , J. Pavlačková , D. Janáčová , and J. Orsavová . 2020. “Chicken Heads as a Promising By‐Product for Preparation of Food Gelatins.” Molecules 25, no. 3: 494. 31979349 10.3390/molecules25030494 PMC7037018

[fsn370651-bib-0025] Gómez‐Guillén, M. , B. Giménez , M. a. López‐Caballero , and M. Montero . 2011. “Functional and Bioactive Properties of Collagen and Gelatin From Alternative Sources: A Review.” Food Hydrocolloids 25, no. 8: 1813–1827.

[fsn370651-bib-0026] Gómez‐guillœn, M. , and P. Montero . 2001. “Extraction of Gelatin From Megrim (*Lepidorhombus boscii*) Skins With Several Organic Acids.” Journal of Food Science 66, no. 2: 213–216.

[fsn370651-bib-0027] Hashim, P. , M. M. Ridzwan , and J. Bakar . 2014. “Isolation and Characterization of Collagen From Chicken Feet.” International Journal of Bioengineering and Life Sciences 8, no. 3: 250–254.

[fsn370651-bib-0028] Kaewdang, O. , S. Benjakul , T. Prodpran , T. Kaewmanee , and H. Kishimura . 2016. “Characteristics of Gelatin Extracted From the Swim Bladder of Yellowfin Tuna (Thunnus Albacores) as Affected by Alkaline Pretreatments.” Journal of Aquatic Food Product Technology 25, no. 8: 1190–1201.

[fsn370651-bib-0029] Kahyaoglu, T. 2008. “Optimization of the Pistachio Nut Roasting Process Using Response Surface Methodology and Gene Expression Programming.” LWT‐ Food Science and Technology 41, no. 1: 26–33.

[fsn370651-bib-0030] Kim, T.‐K. , Y.‐K. Ham , D.‐M. Shin , et al. 2020. “Extraction of Crude Gelatin From Duck Skin: Effects of Heating Methods on Gelatin Yield.” Poultry Science 99, no. 1: 590–596. 10.3382/ps/pez519 PMC7587684 32416845

[fsn370651-bib-0031] Kittiphattanabawon, P. , S. Benjakul , W. Visessanguan , and F. Shahidi . 2010. “Comparative Study on Characteristics of Gelatin From the Skins of Brownbanded Bamboo Shark and Blacktip Shark as Affected by Extraction Conditions.” Food Hydrocolloids 24, no. 2–3: 164–171.

[fsn370651-bib-0032] Lee, S.‐J. , K. H. Kim , Y.‐S. Kim , et al. 2012. “Biological Activity From the Gelatin Hydrolysates of Duck Skin By‐Products.” Process Biochemistry 47, no. 7: 1150–1154.

[fsn370651-bib-0033] Liu, T. , H. Dai , L. Ma , et al. 2019. “Structure of Hyla Rabbit Skin Gelatin as Affected by Microwave‐Assisted Extraction.” International Journal of Food Properties 22, no. 1: 1594–1607.

[fsn370651-bib-0034] Mad‐Ali, S. , S. Benjakul , T. Prodpran , and S. Maqsood . 2017. “Characteristics and Gel Properties of Gelatin From Goat Skin as Affected by Spray Drying.” Drying Technology 35, no. 2: 218–226.

[fsn370651-bib-0035] Mirzapour‐Kouhdasht, A. , F. Sabzipour , M. S. Taghizadeh , and M. Moosavi‐Nasab . 2019. “Physicochemical, Rheological, and Molecular Characterization of Colloidal Gelatin Produced From Common Carp By‐Products Using Microwave and Ultrasound‐Assisted Extraction.” Journal of Texture Studies 50, no. 5: 416–425. 31081544 10.1111/jtxs.12408

[fsn370651-bib-0036] Mohtar, N. , C. Perera , and S. Quek . 2011. “Utilisation of Gelatine From NZ Hoki (*Macruronus novaezelandiae*) Fish Skins.” International Food Research Journal 18, no. 3: 1111–1115.

[fsn370651-bib-0037] Mokrejš, P. , P. Mrázek , R. Gál , and J. Pavlačková . 2019. “Biotechnological Preparation of Gelatines From Chicken Feet.” Polymers 11, no. 6: 1060. 31216750 10.3390/polym11061060 PMC6631408

[fsn370651-bib-0038] Montero, M. , and Ó. G. Acosta . 2020. “Tuna Skin Gelatin Production: Optimization of Extraction Steps and Process Scale‐Up.” CyTA Journal of Food 18, no. 1: 580–590.

[fsn370651-bib-0039] Mukherjee, I. , and M. Rosolen . 2013. “Thermal Transitions of Gelatin Evaluated Using DSC Sample Pans of Various Seal Integrities.” Journal of Thermal Analysis and Calorimetry 114: 1161–1166.

[fsn370651-bib-0040] Muyonga, J. , C. Cole , and K. Duodu . 2004. “Fourier Transform Infrared (FTIR) Spectroscopic Study of Acid Soluble Collagen and Gelatin From Skins and Bones of Young and Adult Nile Perch (*Lates niloticus*).” Food Chemistry 86, no. 3: 325–332.

[fsn370651-bib-0041] Nath, P. , N. Pandey , M. Samota , et al. 2022. “Browning Reactions in Foods.” In Advances in Food Chemistry: Food Components, Processing and Preservation, 117–159. Springer.

[fsn370651-bib-0042] Nik Aisyah, N. , H. Nurul , M. Azhar , and A. Fazilah . 2014. “Poultry as an Alternative Source of Gelatin.” Health and the Environment Journal 5, no. 1: 37–49.

[fsn370651-bib-0043] Norziah, M. , H. Kee , and M. Norita . 2014. “Response Surface Optimization of Bromelain‐Assisted Gelatin Extraction From Surimi Processing Wastes.” Food Bioscience 5: 9–18.

[fsn370651-bib-0044] Nuñez, S. M. , C. Cárdenas , P. Valencia , et al. 2023. “Effect of Adding Bovine Skin Gelatin Hydrolysates on Antioxidant Properties, Texture, and Color in Chicken Meat Processing.” Food 12, no. 7: 1496. 10.3390/foods12071496 PMC10094089 37048317

[fsn370651-bib-0045] Park, J.‐H. , J.‐H. Choe , H.‐W. Kim , et al. 2013. “Effects of Various Extraction Methods on Quality Characteristics of Duck Feet Gelatin.” Korean Journal for Food Science of Animal Resources 33, no. 2: 162–169.

[fsn370651-bib-0046] Perez‐Puyana, V. , F. J. Ostos , P. López‐Cornejo , A. Romero , and A. Guerrero . 2019. “Assessment of the Denaturation of Collagen Protein Concentrates Using Different Techniques.” Biological Chemistry 400, no. 12: 1583–1591. 31125311 10.1515/hsz-2019-0206

[fsn370651-bib-0047] Rafieian, F. , J. Keramat , and M. Shahedi . 2015. “Physicochemical Properties of Gelatin Extracted From Chicken Deboner Residue.” LWT‐ Food Science and Technology 64, no. 2: 1370–1375.

[fsn370651-bib-0048] Ratnasari, I. 2016. “Physico‐Chemical Characterization and Skin Gelatin Rheology of Four Freshwater Fish as Alternative Gelatin Source.” Aquaculture, Aquarium, Conservation & Legislation 9, no. 6: 1196–1207.

[fsn370651-bib-0049] Sadat Hosseini, M. , R. Farahmandfar , A. Motamedzadegan , N. Mollakhalili‐meybodi , and W.‐F. Lai . 2025. “Modifying the Techno‐Functional Characteristics of Quinoa Protein Isolate by Atmospheric Cold Plasma (ACP).” Food Hydrocolloids 169: 111583.

[fsn370651-bib-0050] Saenmuang, S. , S. Phothiset , and C. Chumnanka . 2020. “Extraction and Characterization of Gelatin From Black‐Bone Chicken By‐Products.” Food Science and Biotechnology 29: 469–478. 32296557 10.1007/s10068-019-00696-4 PMC7142178

[fsn370651-bib-0051] Santana, J. C. , R. B. Gardim , P. F. Almeida , et al. 2020. “Valorization of Chicken Feet By‐Product of the Poultry Industry: High Qualities of Gelatin and Biofilm From Extraction of Collagen.” Polymers 12, no. 3: 529. 32121646 10.3390/polym12030529 PMC7182801

[fsn370651-bib-0052] Sarbon, N. M. , F. Badii , and N. K. Howell . 2015. “The Effect of Chicken Skin Gelatin and Whey Protein Interactions on Rheological and Thermal Properties.” Food Hydrocolloids 45: 83–92.

[fsn370651-bib-0053] Shahiri Tabarestani, H. , N. Sedaghat , M. Jahanshahi , A. Motamedzadegan , and M. Mohebbi . 2016. “Physicochemical and Rheological Properties of White‐Cheek Shark ( *Carcharhinus dussumieri* ) Skin Gelatin.” International Journal of Food Properties 19, no. 12: 2788–2804. 10.1080/10942912.2015.1050595.

[fsn370651-bib-0054] Shyni, K. , G. Hema , G. Ninan , S. Mathew , C. Joshy , and P. Lakshmanan . 2014. “Isolation and Characterization of Gelatin From the Skins of Skipjack Tuna (*Katsuwonus pelamis*), Dog Shark (Scoliodon Sorrakowah), and Rohu (*Labeo rohita*).” Food Hydrocolloids 39: 68–76.

[fsn370651-bib-0055] Tümerkan, E. T. A. , Ü. Cansu , G. Boran , J. Mac Regenstein , and F. Özoğul . 2019. “Physiochemical and Functional Properties of Gelatin Obtained From Tuna, Frog and Chicken Skins.” Food Chemistry 287: 273–279. 30857699 10.1016/j.foodchem.2019.02.088

[fsn370651-bib-0056] Widyasari, R. , and S. Rawdkuen . 2014. “Extraction and Characterization of Gelatin From Chicken Feet by Acid and Ultrasound Assisted Extraction.” Food and Applied Bioscience Journal 2, no. 1: 85–97.

[fsn370651-bib-0057] Xu, X. , M. Nikoo , S. Benjakul , et al. 2011. “Characterization of Gelatin From the Skin of Farmed Amur Sturgeon *Acipenser schrenckii* .” International Aquatic Research 3, no. 2: 135–145.

[fsn370651-bib-0058] Yasin, H. , A. S. Babji , and A. S. Norrakiah . 2017. “Modification of Chicken Feet Gelatin With Aqueous Sweet Basil and Lemongrass Extract.” LWT 77: 72–79.

[fsn370651-bib-0059] Zhou, C. , Y. Li , X. Yu , et al. 2016. “Extraction and Characterization of Chicken Feet Soluble Collagen.” LWT ‐ Food Science and Technology 74: 145–153.

